# Supplementary material for: Lithium as a possible therapeutic strategy for Cornelia de Lange syndrome
Source: Cell Death Discov. 2021 Feb 17;7:34. doi: 10.1038/s41420-021-00414-2 (PMC7889653; doi:10.1038/s41420-021-00414-2)
Supplement: Supplementary file 1 — Supplementary Figure Legends [file 41420_2021_414_MOESM1_ESM.docx]

**SUPPLEMENTARY FIGURE LEGENDS**

**Supplementary Figure 1. Brain MRI and CdLS patients**

(A) MRI data distribution of tissue anomalies in CdLS patients. (B) Distribution of genetic variants in the CdLS patient cohort.

**Supplementary Figure 2. CdLS Brain abnormalities in hindbrain-derived structures correlates with cognitive and behavioral alterations**

(A) MRI data distribution in the cohort of CdLS patients. Purple: no MRI (89/155); mauve: MRI negative (35/155); pink: MRI positive (31/155). (B) CNS anomalies and behavioral disabilities. Solid mauve: MRI negative; striped mauve: MRI anomalies. (C) CNS anomalies and behavioral disabilities based on the embryological derivation. Solid mauve: MRI negative; striped mauve: Rhombencephalon derivation; dotted mauve: Prosencephalon derivation. * p<0.05; * vs MRI negative.

**Supplementary Figure 3. Vital counts of lymphoblastoid cell lines**

(A, B) Proliferation assay using Ki-67 was performed to evaluate the viability in CdLS LCLs compared to healthy donors (HD). (A) In standard conditions, the proliferation of patient-derived cells (CdLS H_2_O, white bar) is reduced compared to the proliferation rate of healthy donors (HD H_2_O, white bar). CdLS cell lines (blue striped bar) exposed to LiCl (2.5mM) have an increased proliferation compared with untreated CdLS cells (CdLS H_2_O, white bar) and treated HD cells (blue solid bar). On the axis are reported: the experimental groups (x-axis), and numbers of cells in proliferation at 24 hours of lithium exposure, normalized on water/vehicle (y-axis). Bars express mean ± SEM. (B) Examples of cells in proliferation following Ki-67 immunoassay. Images were taken at 40X, while insets display magnification of the white square (80X). (C-F) Treatments with others WNT pathway activators are shown (HD untreated: dotted bars; treated HD: solid colored bars). Untreated CdLS cells (white bars) are exposed to different concentrations of the compounds (oblique striped bars). Treatments with (C) BIO 0.1 μM, 0.5 μM, 1 μM; (D) IQ-1 5 μM, 10 μM, 20 μM; (E) Deoxycolic acid 5 μM, 100 μM, 250 μM; (F) CHIRR99021 1 μM, 5 μM, 10 μM are represented. On the axis are reported: the experimental groups (x-axis), and numbers of number of live cells at 24 hours of exposure divided by number of live cells at T0, normalized on water/vehicle (y-axis). * p< 0.05, ** p<0.01.
