# Supplementary material for: Lithium as a possible therapeutic strategy for Cornelia de Lange syndrome
Source: Cell Death Discov. 2021 Feb 17;7:34. doi: 10.1038/s41420-021-00414-2 (PMC7889653; doi:10.1038/s41420-021-00414-2)
Supplement: Supplementary file 2 — Supplementary Tables [file 41420_2021_414_MOESM2_ESM.docx]

**SUPPLEMENTARY TABLES**

| **Mouse gene** | **Primer sequence** |
| --- | --- |
| *Cyclin D1 (Ccnd1)* | Forward: CGTGGCCTCTAAGATGAAGGA |
|  | Reverse: CCTCGGGCCGGATAGAGTAG |
| *Actin, beta (Actb)* | Forward: TCCATCATGAAGTGTGACGT |
|  | Reverse: GAGCAATGATCTTGATCTTCAT |
| *18S ribosomal RNA (Rn18s)* | Forward: TTGACGGAAGGGCACCACCAG |
|  | Reverse: GCACCACCACCCACGGAATCG |

**Supplementary table 1.** Primers’ sequences for qPCR analysis (mouse NSCs)

| ***Drosophila melanogaster* gene** | **Primer sequence** |
| --- | --- |
| *Armadillo (arm)* | Forward: TCTGCTGCAACGAAACAACG |
|  | Reverse: CTGCATCCGAAAGATTGCGG |
| *Engrailed (en)* | Forward: TATCGCCGCACTTCAAAAGC |
|  | Reverse: TTTACAGAGCGGTTGCAAGC |
| *Ribosomal protein L32 (RpL32)* | Forward: ACAGGCCCAAGATCGTGAAG |
|  | Reverse: CTTGCGCTTCTTGGAGGAGA |

**Supplementary table 2.** Primers’ sequences for qPCR analysis (*Drosophila*)

| **CdLS LCLs** | **Gene** | **Exon** | **cDNA change** | **Protein change** | **Mutation type** | **Clinical features** | **Reference** |
| --- | --- | --- | --- | --- | --- | --- | --- |
| Sp11 | ***NIPBL*** | 19 | c.4253G>A | p.(G1418E) | Missense | Mild | This work |
| Sp12 | ***NIPBL*** | 4 | c.231-2_231-1delAG | p.(E78Vfs*4) |  | Moderate | This work |
| Sp47 | ***NIPBL*** | - | t(5;15);inv(5p) | - |  | Severe | This work |
| Sp35 | ***SMC1A*** | 2 | c.173del15 | p.(V58_R62del) | In frame deletion | Moderate | Gervasini et al. 2013 (pt #2) |
| 202 | ***SMC1A*** | 15 | c.2351T>C | p.(I784T) | Missense | Moderate | Gervasini et al. 2013 (pt #5) |
| Sp59 | ***HDAC8*** | 10 | c.1022delG | p.(G341Vfs*33) | Frameshift | Mild | This work |

**Supplementary table 3.** Lymphoblastoid immortalized lines from patients, clinical features and related mutations.

| **Human gene** | **Primer sequence** |
| --- | --- |
| *Cyclin D1 (CCND1)* | Forward: CTGGAGGTCTGCGAGGAA |
|  | Reverse: GGGGATGGTCTCCTTCATCT |
| *Glyceraldehyde 3-phosphate dehydrogenase (GAPDH)* | Forward: GAGTCAACGGATTTGGTCGT |
|  | Reverse: TTGATTTTGGAGGGATCTCG |

**Supplementary table 4.** Primers’ sequences for qPCR analysis (Lymphoblastoid cell lines)
