# Supplementary figures and images for: Lithium as a possible therapeutic strategy for Cornelia de Lange syndrome
Source: Cell Death Discov. 2021 Feb 17;7:34. doi: 10.1038/s41420-021-00414-2 (PMC7889653; doi:10.1038/s41420-021-00414-2)

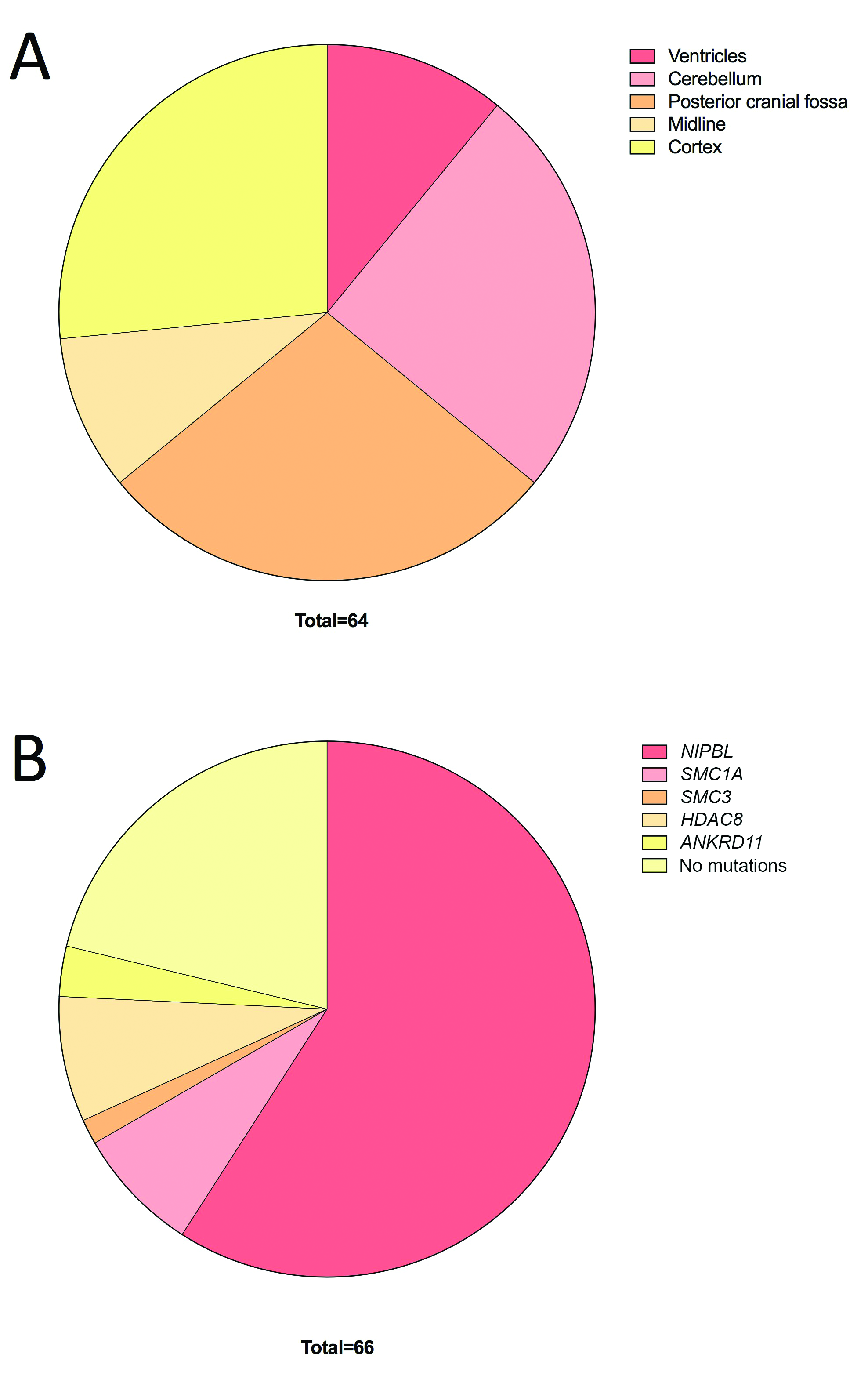

Supplement: Supplementary file 4 — Supplementary Figure 1 [file 41420_2021_414_MOESM4_ESM.tif]

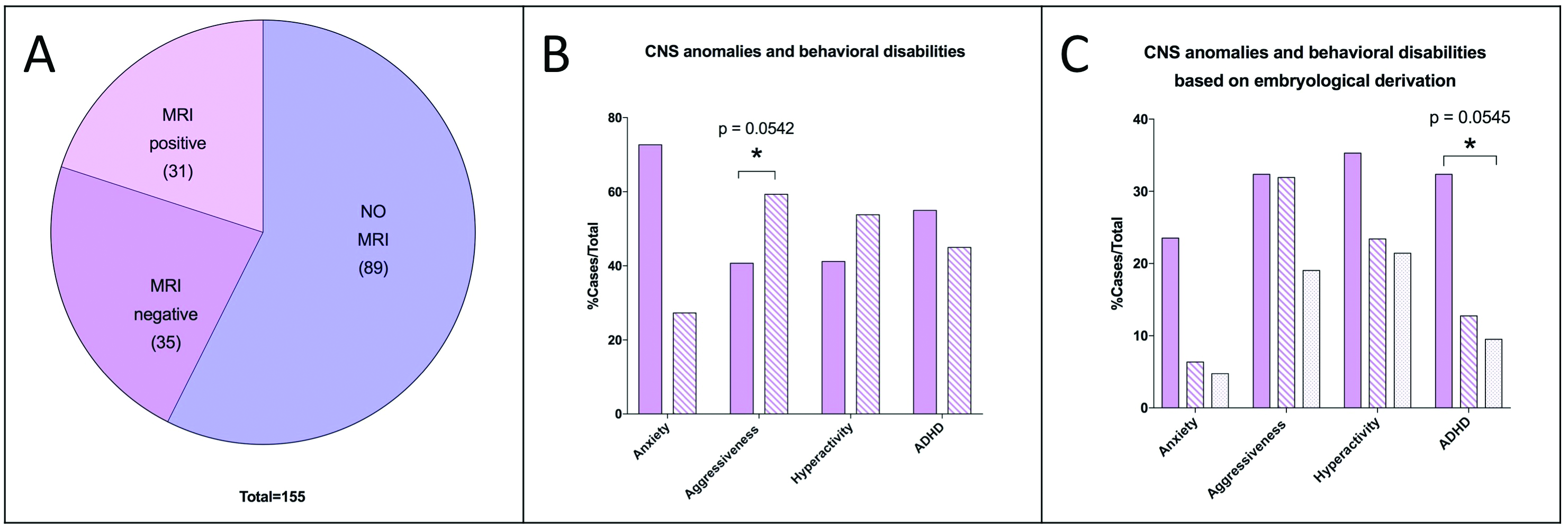

Supplement: Supplementary file 5 — Supplementary Figure 2 [file 41420_2021_414_MOESM5_ESM.tif]

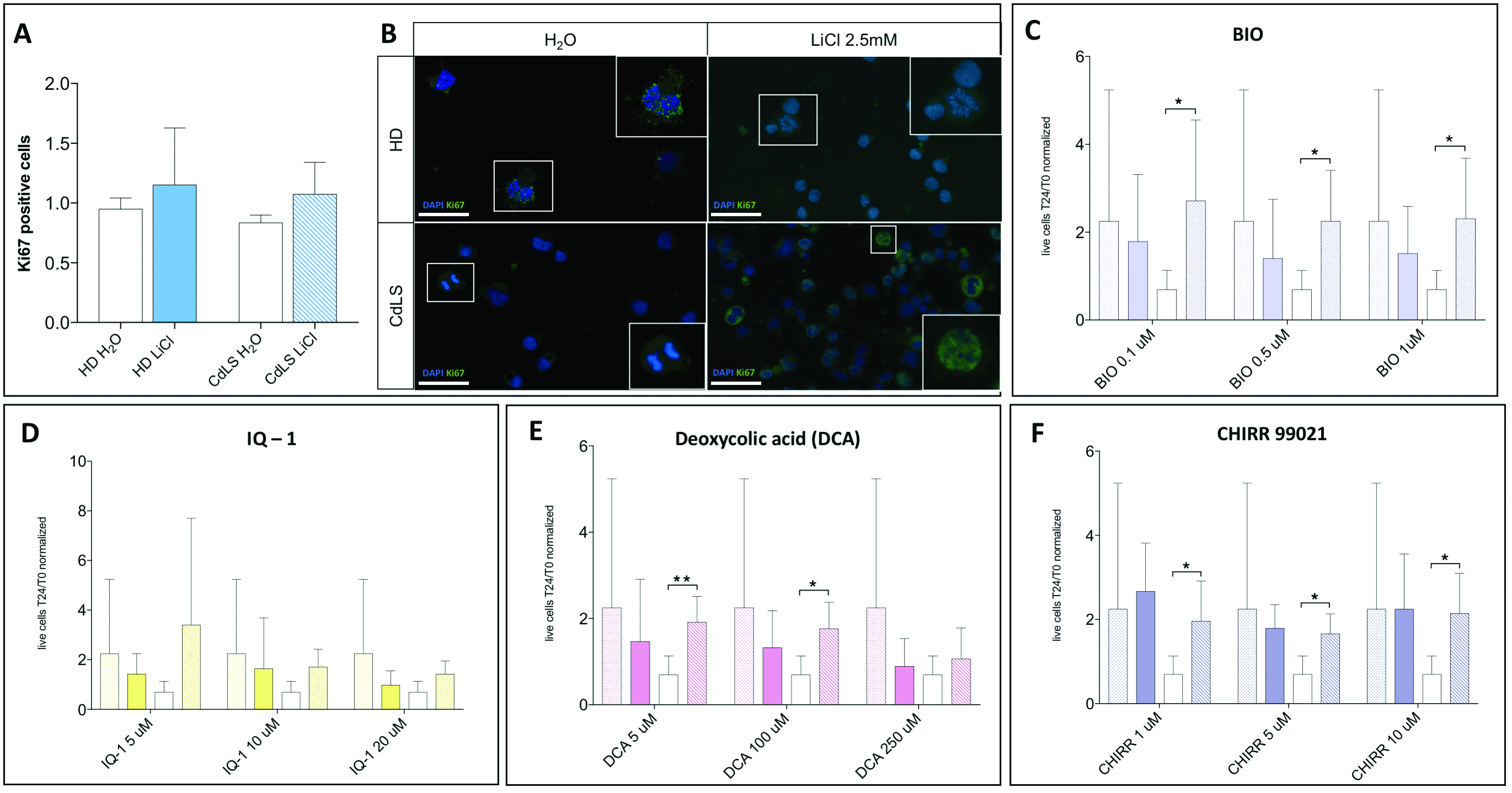

Supplement: Supplementary file 6 — Supplementary Figure 3 [file 41420_2021_414_MOESM6_ESM.tif]
